# A Wide Frequency Scanning Printed Bruce Array Antenna with Bowtie and Semi-Circular Elements †

**DOI:** 10.3390/s20236796

**Published:** 2020-11-27

**Authors:** Zeeshan Ahmed, Patrick McEvoy, Max J. Ammann

**Affiliations:** 1 Antenna and High-Frequency Research Centre, Technological University Dublin, Dublin, Ireland; patrick.mcevoy@tudublin.ie (P.M.); max.ammann@tudublin.ie (M.J.A.); 2 CONNECT—Ireland’s Research Centre for Future Networks and Communications, Westland Row 34, Dublin, Ireland

**Keywords:** meander line antenna, periodic structure, millimeter-wave antenna, frequency scanning antenna, leaky-wave antenna

## Abstract

A printed edge-fed counterpart of the wire Bruce array antenna, for frequency scanning applications, is presented in this paper. The unit-cell of the proposed antenna consists of bowtie and semi-circular elements to achieve wide bandwidth from below 22 GHz to above 38 GHz with open-stopband suppression. The open-stopband suppression enables a wide seamless scanning range from backward, through broadside, to forward endfire. A sidelobe threshold level of −10 dB is maintained to evaluate efficient scanning performance of the antenna. The antenna peak realized gain is 15.30 dBi, and, due to its compact size, has the ability to scan from −64° to 76°.

## 1. Introduction

**Figure 1:**
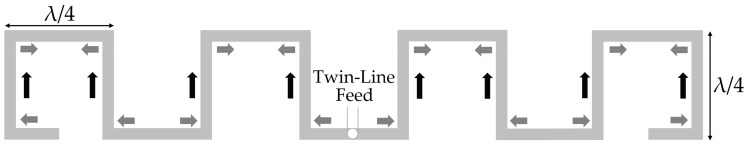
A typical twin-line fed 8-element wire Bruce array antenna (BAA).

In 1931, Edmond Bruce patented the idea of the Bruce array antenna (BAA) in which a long wire antenna was bent in equal and periodic meandered intervals. The antenna was designed for amateur radio applications in which bi-directional broadside radiation and high gain are required. Figure 1 shows a typical BAA fed from the center of the structure using a twin-line feed mechanism. In the figure, the lengths and directions of the arrows are representations of the magnitudes and flow of the current, respectively. The horizontal and vertical segments of the meander line were both kept equal in size, i.e., approximately λ/4 for ham radio applications, except for the last two inward bent segments, which are half the length of the other segments. The currents in the horizontal segments, represented by light grey colored arrows in Figure 1, flow in opposite directions so as to add together destructively, thus cancelling out radiation in ideal circumstances. These horizontal elements are, therefore, considered interconnecting segments. The currents in the vertical segments flow in the same direction, adding constructively in phase to give broadside radiation, which is why these segments are termed radiating elements. The half-length segments, which are bent inward on either ends of the structure, have little to no magnitude of current; therefore, they maintain reasonably low cross-polarized radiation [1]. As the number of radiating elements are added to the structure, the half power beamwidth (HPBW) becomes sharp with the increase in peak realized gain and the radiation pattern in the broadside becomes so compressed and narrow that it can be classified as a highly directive fan-beam radiation pattern. The BAA offers reduced complexity, substantially greater bandwidth than other wire antennas (such as the bobtail curtain and half-square antennas), and, for a relatively low height requirement, it can achieve the maximum possible gain in a given area [2]. Suited to a particular installation, the wire BAA can also be fed at points other than the center, the lengths can be varied to tune the resonant frequency, and while it usually does not require a ground system, an extensive ground system can be deployed under the BAA to mitigate the losses if there is adequate space [2].

The BAA has been around for over a century, but, in spite of its simplicity, modern researchers have overlooked its development and utilization in modern day antenna applications. There are only a handful of ideas proposed to make use of the structure, some of which include Nakano et al.’s concatenation of the Bruce and Franklin antennas’ performance at 12.50 GHz [3], Chen’s twin-line fed slot-type BAA planar equivalent [4], a tri-band mm-wave printed counterpart of BAA [5], and an edge-fed printed BAA [6].

In 1940, W. W. Hansen patented the first waveguide-based leaky-wave antenna (LWA) [7]. Several other researchers later elaborated the concept in their research [8,9,10,11], but it was A. A. Oliner who streamlined the working mechanism in 1984 [12]. In IEEE standard 145-2013, an LWA is defined as “An antenna that couples power in small increments per unit length, either continuously or discretely, from a travelling-wave structure to free space” [13]. LWAs are generally divided into two categories, namely uniform and periodic LWAs [14], the latter of which are widely used in mm-wave frequency regions as well as other scanning applications because of their ability to scan a wider area than the uniform LWAs [15]. Planar periodic LWAs are low-profile, relatively easier to fabricate, and can scan in the backward or the forward endfire direction with a fan-beam radiation pattern with frequency tuning. Several types of LWAs based on a range of technologies have been proposed in the scientific literature, including periodically meandered rampart array [16], sharpening the bends [17], squarely modulated reactance surface (SquMRS) [18], composite right/left-handed structures (CRLH) [19,20], slot or coplanar lines [21], substrate integrated waveguide (SIW) structures [22,23,24,25,26,27], Goubau line structures [24], spoof plasmon transmission line (SSP-TL) structures [25], and periodically loaded microstrip structures [26].

In the case of periodic LWAs, a steep gain-drop is usually observed around the broadside when scanning from the backward to forward endfire, because of which the antenna suffers from pattern degradation. This is because of the presence of the so-called open-stopband (OSB) at which the LWA, which usually supports a traveling-wave, exhibits standing-wave characteristics with equal excitation of the unit-cells. At OSB frequency, the incident power from the unit-cell that is supposed to radiate outwards instead reflects into the source due to the coupling of a contra-directional pair of space harmonics (Floquet modes) [28]. There are numerous periodic LWAs that have either overcome or suppressed this problem. Balanced transmission lines are used in Metamaterial LWAs to enable seamless scanning through the broadside [29,30]. Other than that, SIW structures use shorting vias [31], unequal width in transversal elements of meander lines [16], and non-identical elements in their unit-cells [32]. A lattice-network based TL model [33] has also been reported to suppress the OSB.

This paper presents a modification of planar, edge-fed, periodic array using meandering concept of wire BAA geometry and the suppression of the OSB around the broadside by replacing horizontal and vertical segments with semi-circular and novel bowtie elements, respectively. The unit-cell is the repetitive part of the structure designed at the broadside frequency. The optimizations and simulations were performed using CST Microwave Studio, in which the dielectric and metallization losses were considered. Finally, the prototype antenna was fabricated and measured responses were compared against the simulated results, from which a satisfactory agreement was attained.

## 2. Unit-Cell and Antenna Geometry

**Figure 2:**
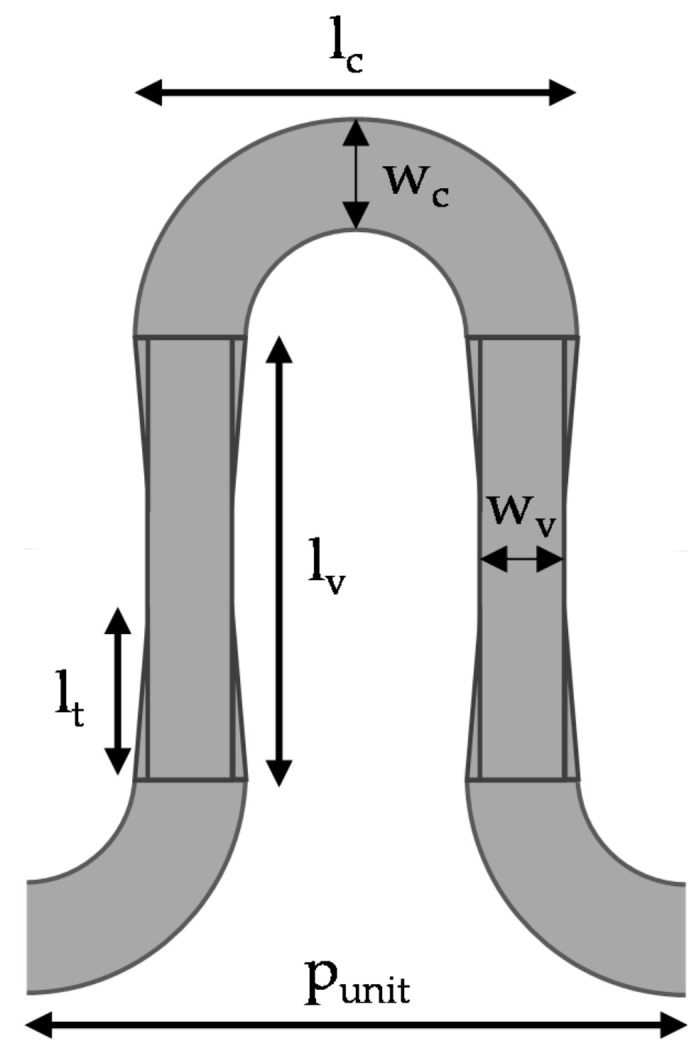
Geometry of the modified BAA unit-cell with vertical bowtie and horizontal semi-circular segments printed on a 0.254 mm thick grounded Arlon DiClad 880.

The configuration of the unit-cell of modified printed BAA is shown in Figure 2. The vertical and horizontal segments of the meander-line BAA antenna, shown in Figure 1, were replaced with bowtie and semi-circular elements, respectively. Either ends of the bowtie had the same width as the semi-circular segment, i.e., *w_c_*. Compared to the BAA, the meandered segments’ lengths are approximately λ/4, but in the mm-wave region, this corresponds to a very small size which gives rise to coupling between the vertical segments; therefore, these lengths are varied. However, the length and diameter of the unit-cell, *l_v_* and *l_c_,* respectively, are kept the same, as shown in Table 1. The periodic unit-cell’s dominant mode does not radiate on its own because of its slow-wave characteristics; the free space wavenumber, *k_0_*, is less than the phase constant (*β* > *k*_0_). Floquet’s theorem states that as the unit-cells are combined in series, the periodicity introduces an infinite number of Floquet modes in a leaky-wave structure. These Floquet modes are represented by phase constant *β_n_* [14].

(1) $$\beta_{n}p_{unit}=\;\beta_{0}p_{unit}+2\pi n;\;n=0,\;\pm 1,\;\pm 2,\;\pm 3$$

where *p_unit_* is the period of the unit-cell defined by 4 × (*l_c_* − *w_c_/*2), *n* is the *n*th number space harmonic, and *β_0_* is the phased constant of the dominant mode of the now modulated and uniform waveguide. From Equation (1) the *β_0_* is slow-wave, but the structure is designed in a way that the other modes are fast. In order to scan a single beam in a directive manner, the first space harmonic, i.e., *n* = −1, is substituted into Equation (1) and is written as

(2) $$\beta_{-1}p_{unit}=\;\beta_{0}p_{unit}-2\pi$$

The scanning direction of the periodic LWA can be expressed using [14]

(3) $$\sin\theta_{m}=\;\frac{\beta_{-1}}{k_{0}}$$

where *θ_m_* is the maximum beam angle deviation from the broadside and *k_0_* is the free space wave number. Subsequently, the beamwidth is given by

(4) $$\Delta \theta \;\approx \;\frac{1}{\left(\frac{L}{\lambda_{0}}\right)\cos\theta_{m}}$$

where *L* is the overall length of the leaky-wave antenna structure.

**Figure 3:**
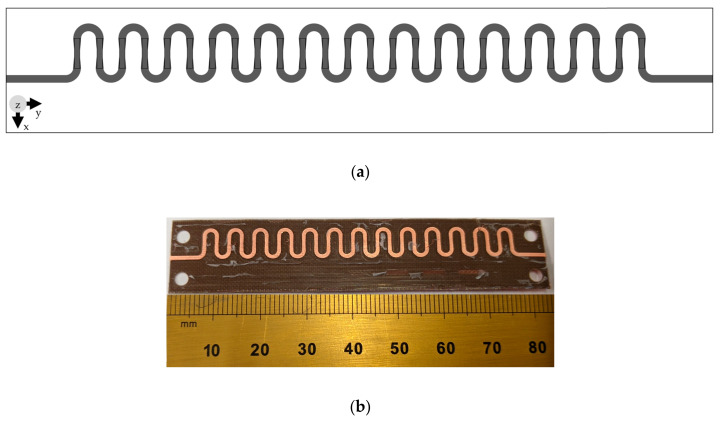
Top view of the proposed periodic 13 unit-cell antenna fabricated on 0.254 mm thick grounded Arlon DiClad 880 substrate. (**a**) Geometry. (**b**) Prototype.

The geometry of the periodic modified BAA antenna, with vertical bowtie and horizontal semi-circular segments, is presented in Figure 3a. As is the case with linear arrays, the addition of unit-cells in series increases the gain and decreases the beamwidth along the length of the antenna, but a large number of unit-cells prevents the increase in gain due to the lower power delivered to the last unit-cells. Thirteen unit-cell elements, presented in Figure 2, were connected in series, and the configuration presented in Table 1 was used for simulation and prototyping of the structure. The periodic modulation of the geometry assisted with radiation along the length of the antenna. The structure was fed using transmission line of length 6.18 mm and width 0.76 mm; the last unit-cell element was terminated using a similar transmission line and another 50 Ω port that acted like a resistor to avoid reflections. Arlon DiClad 880 substrate, with a thickness of 0.254 mm, ε_r_ = 2.2 and tan δ = 0.0009, was used to fabricate the prototype presented in Figure 3b; the measurements were performed using a Rhode and Schwarz Vector Network Analyzer (ZVA40). The overall dimensions of the antenna were 83.60 × 18.0 × 0.254 mm^3^.

## 3. Parametric Analysis

**Figure 4:**
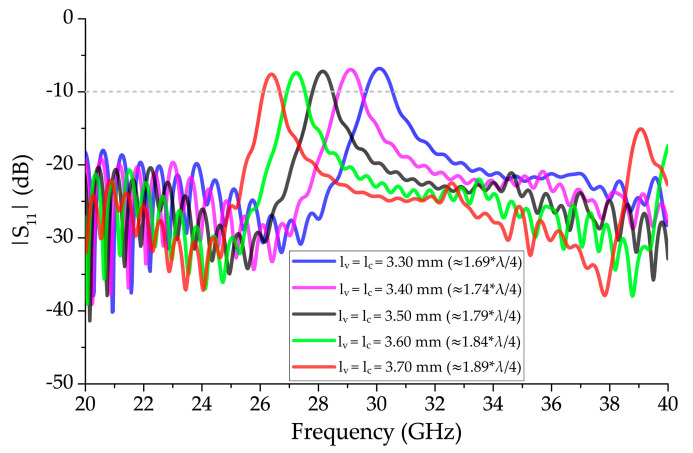
Effect of varying length, *l_v_*, and diameter, *l_c_*, simultaneously for a 13 unit-cell element periodic structure with *w_v_* = 0.76 mm unit-cell segments on |S_11_|.

Figure 4 shows the effects on |S_11_| for the structure with 13 unit-cell elements presented in Figure 3 by simultaneously varying *l_v_* and *l_c_* with *w_v_* = 0.76 mm, without the vertical bowtie element. The multiplying factor of the wire BAAs, λ/4, was increased to avoid unwanted resonances in the mm-wave region, due to close separation distance at λ/4 between vertical elements; it varied from 1.69 × λ/4 to 1.89 × λ/4 (3.30 mm to 3.70 mm). With this configuration, mismatching was observed around the broadside frequencies, for which |S_11_| > −10 dB indicated the presence of OSB. Additionally, an increase in *l_v_* and *l_c_* by 0.1 mm tuned down the OSB mismatched region by approximately 1 GHz. The |S_11_| above and below the OSB, between 20 GHz and 40 GHz, remained less than −10 dB.

**Figure 5:**
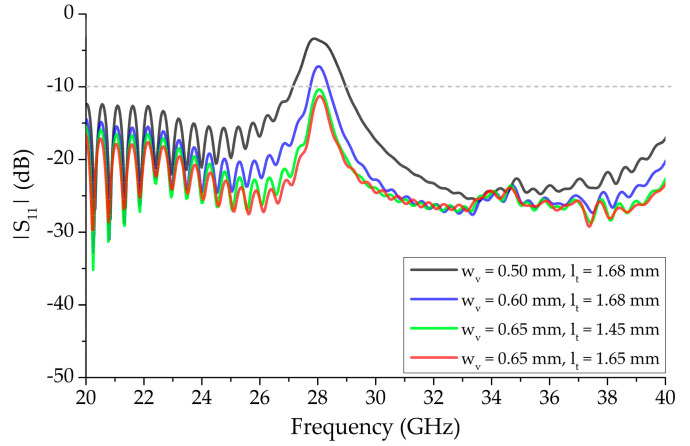
Effect of varying vertical bowtie segment parameters, *w_v_* and *l_t_*, of 13 unit-cell element periodic structure independently on |S_11_| while the other parameters remain the same as Table 1.

The effect of parameters associated with the vertical bowtie segments, *w_v_* and *l_t_*, of the 13 unit-cell structure, on |S_11_|, is shown in Figure 5. The other parameters, presented in Table 1, remain unchanged. A noticeable improvement in impedance matching, around the OSB region, was observed as the width, *w_v_*, was increased from 0.50 mm to 0.60 mm, with *l_t_* fixed at 1.68 mm, in which |S_11_| improved from −7.24 dB to −3.54 dB at 28.0 GHz. In the other case, where *w_v_* was kept constant at 0.65 mm, the variation in *l_t_* from 1.45 mm to 1.65 mm improved the impedance matching significantly without any upward or downward tuning of frequency.

**Figure 6:**
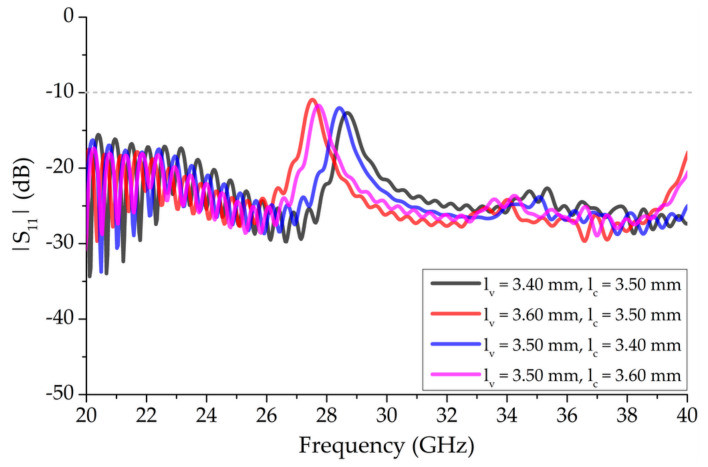
Effect of varying length, *l_v_*, and diameter, *l_c_*, independently of 13 unit-cell element periodic structure with fixed *w_v_* = 0.65 mm bowtie segments on |S_11_|.

Figure 6 shows the effect of independently varying *l_v_* and *l_c_* with the fixed *w_v_* = 0.65 mm bowtie vertical unit-cell segment on |S_11_|. The frequency was tuned down by approximately 1.15 GHz and the impedance matching deteriorated when *l_v_* was increased by 0.20 mm, between 3.40 mm and 3.60 mm, and *l_c_* was kept constant at 3.50 mm. A downward shift in frequency of 0.70 GHz was observed when *l_v_* was fixed at 3.50 mm and *l_c_* was increased from 3.40 mm to 3.60 mm, with a minute effect on impedance matching.

**Figure 7:**
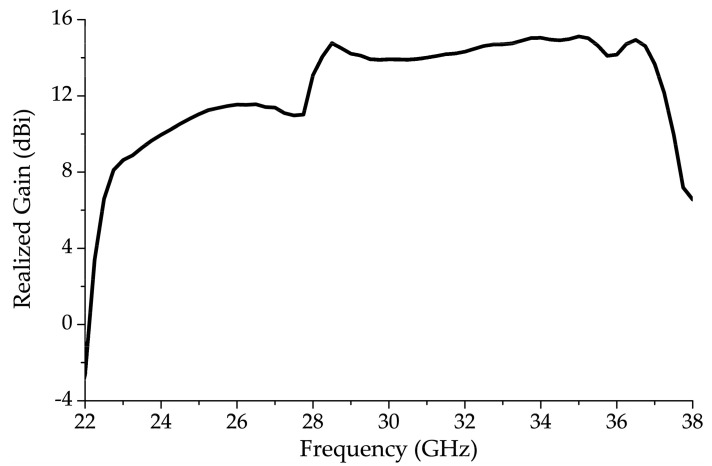
Realized gain of 1-D periodic BAA-LWA with *l_v_* = *l_c_* = 3.50 mm and *w_v_* = 0.76 mm unit-cell showing gain degradation around 28.0 GHz.

Figure 7 shows the realized gain of the 13 unit-cell antenna structure without the vertical bowtie element at between 22 GHz and 38 GHz with *l_v_* = *l_c_* = 3.50 mm and *w_v_* = 0.76 mm. The gain rose gradually between 23.0 GHz and 26.0 GHz and, while still under 12.0 dBi at 26.0 GHz, a sharp gain drop was observed around 28.0 GHz. The realized gain around 28.0 GHz was considerably less than the realized gain in the rest of the forward endfire region. This is consistent with the OSB region, identified in Figure 4, where |S_11_| > −10 dB.

## 4. Results and Discussion

**Figure 8:**
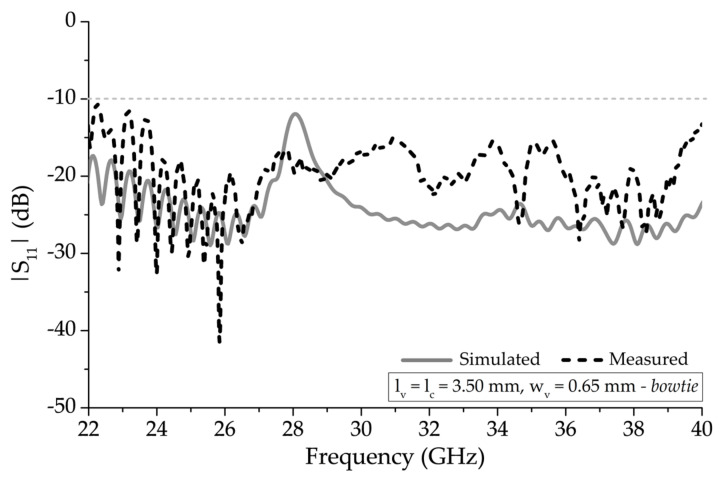
Simulated and measured |S_11_| of 13 unit-cell element periodic structure with bowtie vertical unit-cell segments.

Figure 8 shows the simulated and measured response of the |S_11_| of the 13-element structure with the bowtie vertical segment unit-cells that were shown in Figure 2 and the parameters presented in Table 1. The mismatched OSB frequency range, for when *l_v_* and *l_c_* is 3.50 mm (~1.79 × λ/4) and *w_v_* = 0.76 mm as presented in Figure 4, improved without any frequency tuning, resulting in the mitigation of the OSB. The |S_11_|, at 28.0 GHz, improved to −12.01 dB with the vertical bowtie element, compared to −7.73 dB without the bowtie element. With the bowtie element, the |S_11_|was ≤ −10 dB from below 22.0 GHz and above 40.0 GHz with a fractional bandwidth of more than 67%.

**Figure 9:**
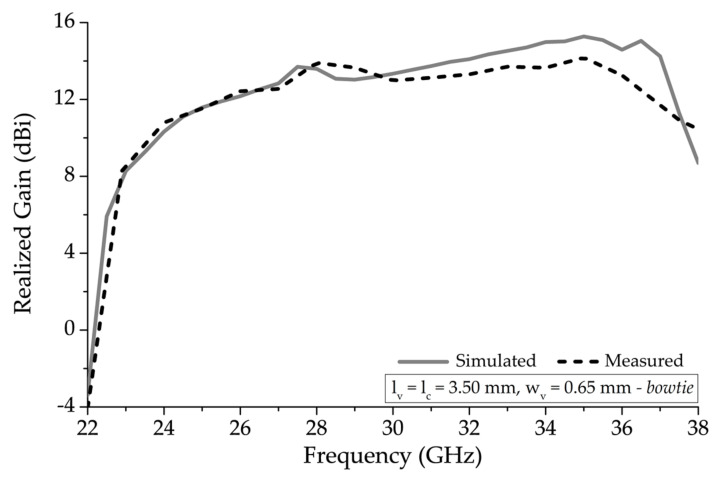
Simulated and measured realized gain comparison of a 13 unit-cell element periodic structure with *w_v_* = 0.76 mm and a modified bowtie vertical unit-cell segments.

Figure 9 shows the realized gain plot of the antenna structure with the modified bowtie unit-cell shown in Figure 2. The sharp decline in realized gain around 28.0 GHz region, shown in Figure 7, considerably improved with this arrangement using the parameters presented in Table 1. The gain profile gradually increased around 23.0 GHz, and onwards, with peak realized gain of 15.30 dBi at 35.0 GHz.

**Figure 10:**
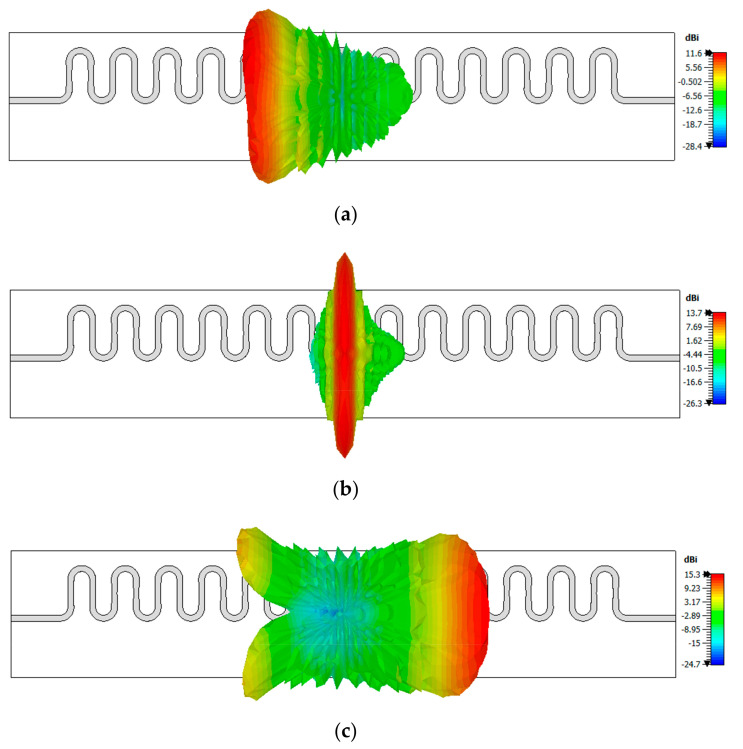
Three-dimensional radiation pattern visualizing scanning at (**a**) 24.0 GHz, (**b**) 28.0 GHz, and (**c**) 35.0 GHz.

**Figure 11:**
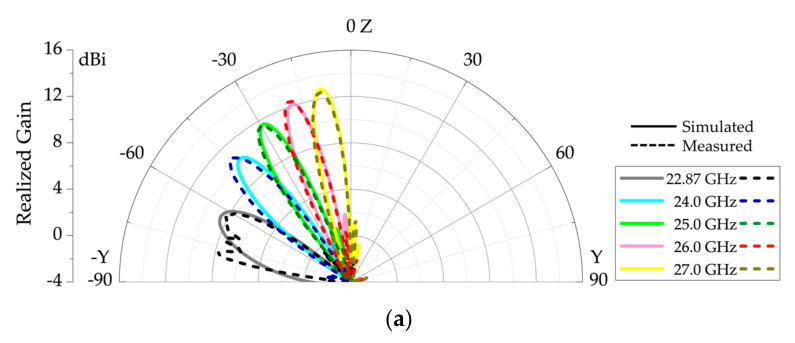

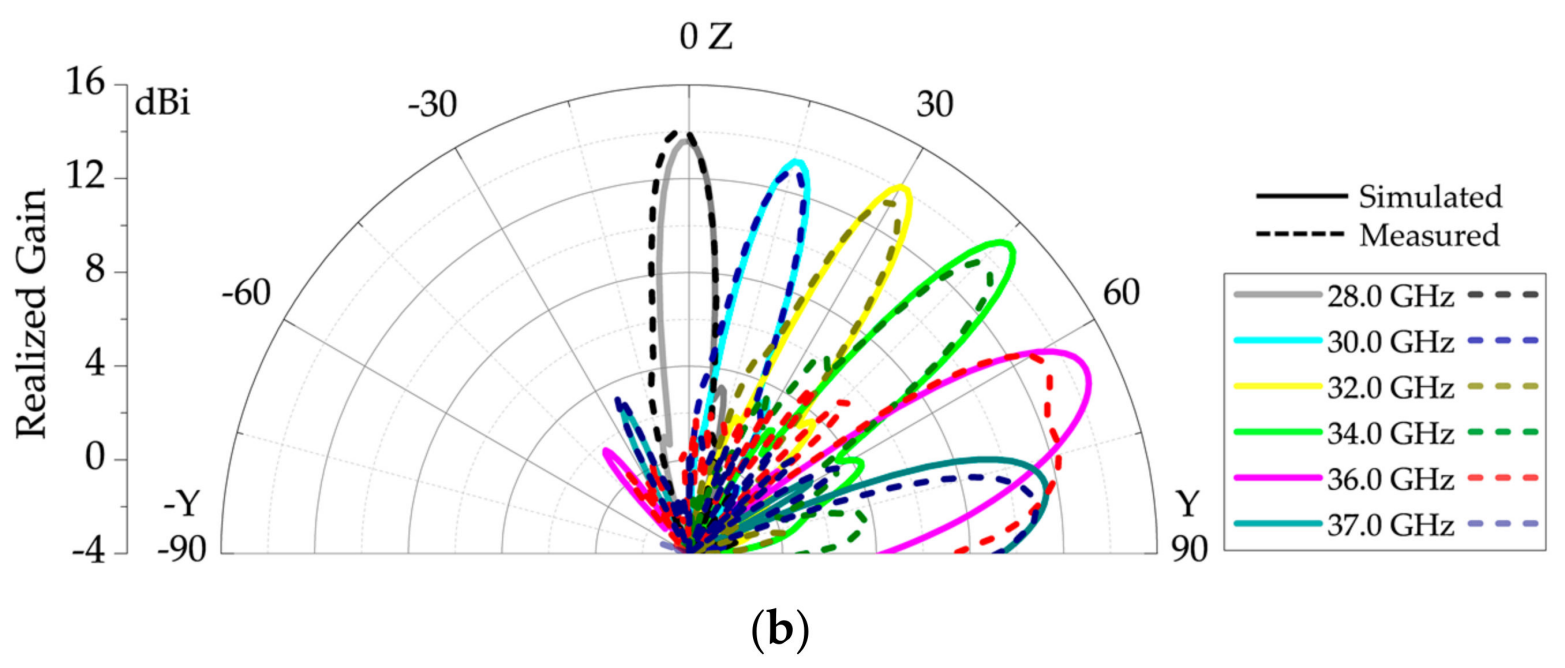
Scanning range of the proposed 1-D periodic modified BAA-LWA with bowtie and semi-circular unit-cell (**a**) backward quadrant and **(b**) forward quadrant.

The 3D radiation patterns for the simulation of the 13-element structure, with bowtie and semi-circular unit-cell, are presented for backward endfire, broadside and forward endfire regions in Figure 10a–c, respectively. The patterns, at 24.0 GHz, 28.0 GHz and 35.0 GHz, showed a fan-beam scanning with an increase in frequency and radiation angles of −42°, 0° and 56°, respectively. The scanning range of the proposed antenna is presented in Figure 11. Figure 11a shows the scanning range from backward endfire approaching towards the broadside. The antenna scanned from −64° at 22.87 GHz. Figure 11b shows the scanning range from the broadside to the forward endfire. The antenna scanned seamlessly through the broadside, due to the mitigation of OSB, until 76°, i.e., 37.0 GHz.

**Figure 12:**
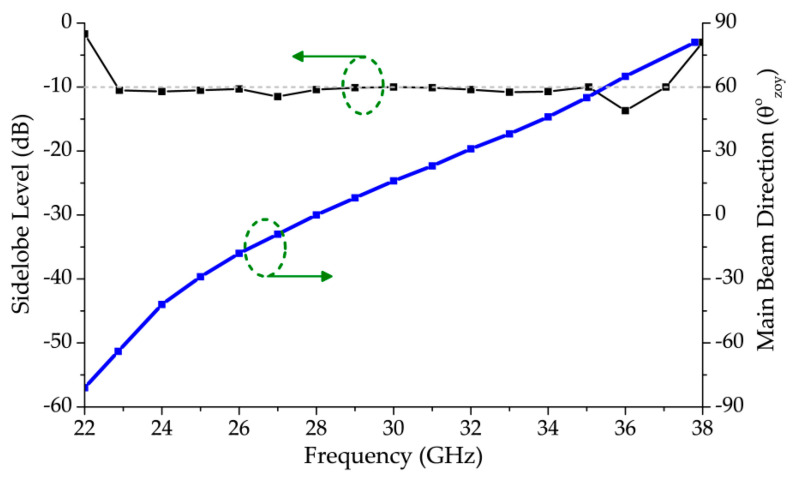
Sidelobe level and main beam direction in the yz-plane of the proposed LWA.

Figure 12 shows the main beam direction and sidelobe level (SLL), in the yz-plane, of the proposed antenna. From Figure 8, it can be seen that the antenna has wide bandwidth below 22.87 GHz and above 37.0 GHz, but these frequencies are not considered as part of the scanning range in Figure 11 because an SLL threshold of −10 dB is maintained to efficiently define the scanning region which. As the mainlobe of the scanning range approaches forward endfire after 76°, the rise in the antenna’s backlobe and the increase in SLL makes it unsuitable to scan in a single direction as efficient as the rest of the considered bandwidth.

**Figure 13:**
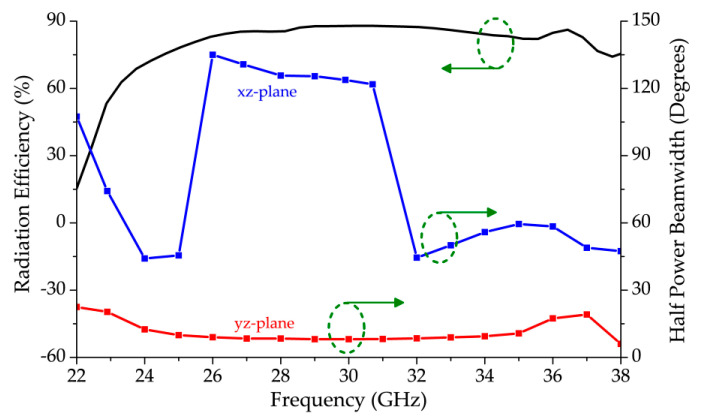
Radiation efficiency and half power beamwidth (HPBW) plots of the proposed LWA.

Figure 13 shows the radiation efficiency of the proposed antenna array. The antenna had more than 60% radiation efficiency throughout the scanning range, and, between 25.0 GHz and 37.0 GHz, the efficiency was more than 80%. The HPBW in both xz and yz-planes, across the entire scanning range, is also shown in the figure. As the beam approaches broadside from backward endfire, the HPBW in xz-plane increased and stabilized before dropping again as it approached forward endfire shown in Figure 10c. From the yz-plane, it can be seen that the antenna had a narrow radiation beam throughout the scanning range which can be classified as fan-beam radiation pattern.

The performance of the proposed antenna’s scanning characteristics was compared with other antennas in the scientific literature and is presented in Table 2. The proposed antenna had wider bandwidth and scanning range along with the peak realized gain than [34,35,36,37] and in [35], only the SLL at the broadside is mentioned. There is no mention of SLL threshold for efficient scanning in any articles except [6,27,38,39]. The presented antenna had better scanning range than the first continuous scanning range of [6]. In [39], although the realized gain and bandwidth are better, it has overall dimensions of 133 × 93 × 21 mm^3^, with a narrower scanning range; it is also difficult to fabricate geometry compared to the presented structure. If not for the SLL ≤ −10 dB threshold maintained throughout this work, the proposed antenna may have had a wider scanning range due to the available bandwidth below 22.87 GHz in the backward endfire and above 37.0 GHz in the forward endfire. The proposed antenna can thus be used for 28 GHz 5G and Ka-Band millimeter-wave imaging applications [40].

## 5. Conclusions

A wide backward to forward endfire scanning leaky-wave antenna is proposed in this paper, as well as a discussion of the results. The initial concept to design the unit-cell of the antenna is taken from meandered wire Bruce array antenna and transformed to printed geometry. The horizontal and vertical segments of the meandered unit-cell were replaced with semi-circular and bowtie segments, respectively, of which the latter assists in the mitigation of the open-stopband at broadside. The length and diameter of both vertical and horizontal segments, respectively, are kept equal at 3.50 mm. The proposed antenna has a wide operational bandwidth from below 22.0 GHz to above 38.0 GHz; however, an SLL threshold of −10 dB was enforced to define an efficient scanning range between 22.87 GHz and 37.0 GHz. The 13 unit-cell periodic antenna has a compact size, offers a scanning range between −64° to 76°, and has peak gain of 15.30 dBi.

## Figures and Tables

**Table 1:** Table of parameters of unit-cell.

Parameter	Size (mm)
*l_c_* = *l_v_*	3.50
*l_t_*	1.68
*w_c_*	0.76
*w_v_*	0.65
*p_unit_*	5.48

**Table 2:** Table of performance comparison between proposed LWA and LWAs in literature.

Ref.	Antenna Type	$\mathrm{Total}\;\mathrm{Length}\;(\lambda_{0})$	Bandwidth (GHz)	Scanning Range	Realized Gain (dBi)	SLL Threshold (dB)
[6]	Periodic dual BAA	6.80	18.0–38.63	−38° to 54°	10.60–16.44	10
				−32° to−5°	16.20–18.61	
[16]	Periodic microstrip	3.68	3.70–6.80	55° to 63°	10.0 (Peak)	N/A
[20]	Multilayered CRLH TL	11.58	20.0–30.0	−25° to 50°	10.0–14.0	N/A
[27]	Half-mode SIW	≈9.30	13.50–16.50	−30° to 30°	10.0 (Peak)	–10
[34]	Periodic CRE	N/A	13.0–19.45	−48° to 35°	≈12.0–14.0	N/A
[35]	Periodic microstrip	7.48	20.0–29.0	−50° to 45°	12.20 (Peak)	−13 (Broadside)
[36]	Metasurface LWA	15.85	15.50–16.20	47° to 7°	15.10 (Peak)	N/A
[37]	* Periodic HW-MLWA	3.99	4.40–8.80	144° to 41°	≈1.0–8.0	N/A
[38]	Periodic combline	29.49	8.0–11.40	−25° to 10°	21.0 (Peak)	−10
[39]	WG-CTS	15.08	26.0–42.0	−56° to 2°	≈22.90–29.20	−12.6
This Work	Bowtie and semi-circular periodic BAA	7.80	22.87–37.0	−64° to 76°	8.57–15.30	−10

CRE—complimentary radiation elements; TL—transmission line; HW-MLWA—half width microstrip LWA; WG—waveguide; CTS—continuous transverse stub; N/A—not available; * OSB from 5.30 GHz to 6.20 GHz.
